# Plasma microRNA-133a is a new marker for both acute myocardial infarction and underlying coronary artery stenosis

**DOI:** 10.1186/1479-5876-11-222

**Published:** 2013-09-23

**Authors:** Feng Wang, Guangwen Long, Chunxia Zhao, Huaping Li, Sandip Chaugai, Yan Wang, Chen Chen, Dao Wen Wang

**Affiliations:** 1 The Institute of Hypertension and Department of Internal Medicine, Tongji Hospital, Tongji Medical College, Huazhong University of Science and Technology, Wuhan, People’s Republic of China; 2 Department of Internal Medicine, Tongji Hospital, Tongji Medical College, Huazhong University of Science and Technology, 1095# Jiefang Ave, Wuhan 430030, PR China

**Keywords:** Biomarker, CHD, Circulating miRNA

## Abstract

**Background:**

Previous study demonstrated that miR-133a was released into blood from injured myocardium in cardiovascular diseases. However, the dynamic change of circulating miR-133a level in the early phase of acute myocardial infarction (AMI) and the correlation between miR-133a and severity of coronary stenosis in coronary heart disease (CHD) patients are not clear.

**Methods and results:**

Three different cohorts (including 13 AMI patients, 176 angina pectoris patients and 127 control subjects) were enrolled to investigate the expression levels of circulating miR-133a in patients with myocardial ischemia and also the relationship between plasma miR-133a and severity of coronary stenosis. Plasma miR-133a levels of participants were examined by real-time quantitative PCR. Simultaneously, plasma cardiac troponin I (cTnI) concentrations were measured by ELISA assays. The results showed that circulating miR-133a level was significantly increased in AMI patients in time-dependent manner, and achieved a 72.1 fold peak at 21.6 ± 4.5 hours after the onset of AMI symptoms and exhibited a similar trend to plasma cTnI level. We also found that plasma miR-133a levels were higher in CHD patients than control group. Importantly, the levels of circulating miR-133a positively correlated with the severities of the coronary artery stenosis. Receiver operating characteristic (ROC) analysis revealed that circulating miR-133a had considerable diagnostic accuracy for CHD with an AUC of 0.918 (95% confidence interval 0.877-0.960).

**Conclusions:**

Circulating miR-133a may be a new biomarker for AMI and as a potential diagnostic tool. And increased miR-133a level may be used to predict both the presence and severity of coronary lesions in CHD patients.

## Introduction

Acute myocardial infarction (AMI) is the worst acute syndrome of coronary heart diseases (CHD) with high morbidity and mortality. An early and correct diagnosis is critical for providing appropriate therapy to improve the survival rate and prognosis [1]. Blood biomarker cardiac troponin I (cTnI) is widely used in clinical practice as the gold standard for diagnosing acute myocardial infarction [2], but plasma cTnI concentrations may be falsely elevated in certain cardiac as well as non cardiac diseases such as severe heart failure, atrial fibrillation, chronic kidney disease, severe sepsis and septic shock [3-6]. Therefore, it is necessary to search novel biomarkers with high sensitivity and specificity for early diagnosis of AMI.

MicroRNAs (miRNAs) are endogenous small non-coding RNAs with 21-25 nucleotides in length. By pairing with the 3’ UTR of target mRNAs, miRNAs can regulate protein-coding genes at the posttranscriptional level via degradation of mRNAs or repression of protein translation [7]. At present, About 700 human miRNAs have been identified, and most of them were found to be tissue-/cell-specific [8]. Mounting evidences suggest that miRNAs play crucial roles in various physiological and pathologic processes, and the dysfunctions of miRNAs are associated with various diseases and pathophysiologies [9-11]. Recently, studies showed that miRNAs are abundantly present in body fluid and can be used as biomarkers for some diseases [12-14]. MiR-133a is a muscle specific-miRNA and is expressed abundantly in myocardial cells [15-17]. It has been established that miR-133a plays important roles in myogenesis, cardiac development and hypertrophy [18-23]. Previous studies demonstrated that miR-133a had a low level presence in the plasma of healthy people [15], and it was expressed differentially in different cardiovascular diseases [15,24]. Recently, it has been reported that the elevated miR-133a is released into peripheral circulation from the injured myocardium after Ca^2+^ stimulation [25]. Although these studies demonstrated that the expression of circulating miR-133a increased in patients with CHD (including AMI and angina pectoris) and circulating miR-133a can be used as a marker for cardiomyocyte death, few clinical studies have reported on the dynamic change in circulating miR-133a level in the early phase of AMI, and also the correlation between miR-133a concentration and the severity of coronary stenosis in CHD patients is not clear.

In the present work, we aimed to confirm the role of plasma miR-133a as a biomarker for CHD, especially for AMI. Furthermore, we investigated the correlation between the levels of circulating miR-133a and the Gensini score (a numerical value for assessment the severity of coronary artery stenosis) in coronary heart disease patients.

## Materials and methods

### Characteristics of patients

Experiments were conducted in accordance with the Declaration of Helsinki. Three cohorts participated in this study.

The first cohort included 13 patients of AMI and 27 healthy volunteers. The inclusion criteria for AMI patients were based on the third Universal Definition of Myocardial Infarction [26]. Briefly, AMI patients were clinically diagnosed by the following criteria: 1) acute ischemic chest pain within 24 hours; 2) electrocardiogram change of acute myocardial infarction (pathological Q wave, ST-segment elevation or depression); 3) plasma cTnI > 0.1 ng/mL. The initial blood sample (denoted by T0) was collected immediately after the AMI patient was admitted to Tongji hospital. Other 5 subsequent blood samples were obtained at 4, 12, 24, 48, 72 hours after the first collection, denoted by 4 h, 12 h, 24 h, 48 h and 72 h, respectively. The second cohort included 22 CHD patients with chest pain having single lesion of the left anterior descending coronary artery and 8 non-CHD patients with negative results of coronary angiography. The third cohort contained 246 patients with acute chest pain. Further, coronary angiography showed that 154 of them were CHD patients with complex lesions of coronary artery, and the remaining 92 patients were non-CHD patients with no coronary artery stenosis. A single blood sample from each participant in both second and third cohorts was obtained immediately after admission, and coronary angiography was used to confirm CHD and define the coronary artery lesions. Blood samples were collected via venous puncture. After isolation by centrifugation, the plasma were transferred to RNase-free tubes and stored at -80°C until further processing.

Participants were selected from inpatients or outpatients departments of Tongji hospital between October 2009 and June 2011 in Wuhan, China. The study was approved by the Medical Ethics Committee in Tongji Hospital and written informed consents were obtained from all the participants.

### RNA isolation

Total RNAs were isolated by TRIzol LS Reagent (Invitrogen) according to the manufacturer’s protocol as described previously [27]. In brief, total RNA was purified from 500 μL of plasma and ultimately eluted into 25 μL of RNase-free water.

### Detection and quantification of miRNAs by real-time PCR

Two microgram of total RNA was reverse-transcribed using Transcript First-strand cDNA synthesis SuperMix (TransGen Biotech, Beijing, China) according to the manufacturer’s protocol. The Bulge-Loop™ miRNA qRT-PCR Detection Kit (Ribobio Co., Guangzhou, China) and SYBR Green PCR SuperMix Kit (TransGen Biotech, Beijing, China) were used in real-time PCR for examining the relative quantification of miR-133a according to the manufacturer’s protocol with the Rotor-Gene 6000 system (Corbett Life Science, QIAGEN, Hilden, Germany), and U6 was measured as endogenous control for normalizing the data of experimental qRT-PCR. Each specimen was measured in triplicate. The threshold cycle (Ct) was defined as the fractional cycle number at which fluorescence exceed the threshold. In our experiment the detection limit of Ct value was defined as 40. The Ct values from qRT-PCR assays over 40 were treated as 40 [15,25,28,29].

### Cardiac troponin I determination

The concentrations of cardiac Troponin I (cTnI) were measured by the Human Troponin I ELISA kit (Abnova, Taiwan) according to manufacturer’s protocol.

### Statistical analysis

Real-time PCR assays were analyzed by 2^-ΔΔct^ method, which is a widely used method to present relative gene expression by comparative Ct. All the data of patients’ clinical characteristics are described as mean ± SD, and the other data are described as mean ± SEM. The data of miR-133a and cTnI were analyzed by the Kolmogorov-Smirnov test to examine whether they followed the normal distribution. If the data fit the normal distribution, then student’s *t* test and the ANOVA are used. Otherwise, Mann–Whitney *U* test and two-tailed Kruskal-Wallis tests are performed. In this study, both the data of miR-133a and cTnI were found to follow the normal distribution by the Kolmogorov-Smirnov test and hence student’s *t* test and ANOVA were used. Categorical variables were compared by *χ*^2^ test. The correlation analyses were determined by linear regression analysis. The receiver operating characteristic (ROC) curve was used to assess the predictive power for diagnosing CHD. Multiple logistic regression analysis was carried out to investigate whether miRNA-133a was an independent predictor of CHD after adjustment for relevant co-variants (including age, sex, smoking and cardiovascular risk factors hypertension, diabetes, hyperlipidemia etc.) as previously described [30]. All statistical analyses were accomplished by using SPSS 17.0 software, and the cutoff point of statistical significance was set at p < 0.05 (two-sided).

## Results

### The real-time RT-PCR (qRT-PCR) assay for miRNA quantification

To ensure the method of qRT-PCR assay for miR-133a quantification is viable and suitable, the amplification curves for both miR-133a and U6 were provided (Additional file 1: Figure S1A and B). To verify primer specificities, melting curve analyses (Additional file 1: Figure S1C and D) and agarose gel electrophoresis images (Additional file 1: Figure S2A and B) were performed, the RNAs extracted from mouse heart and brain were treated as positive and negative control, respectively.

### The pattern of plasma miR-133a levels in acute myocardial infarction

**Figure 1:**
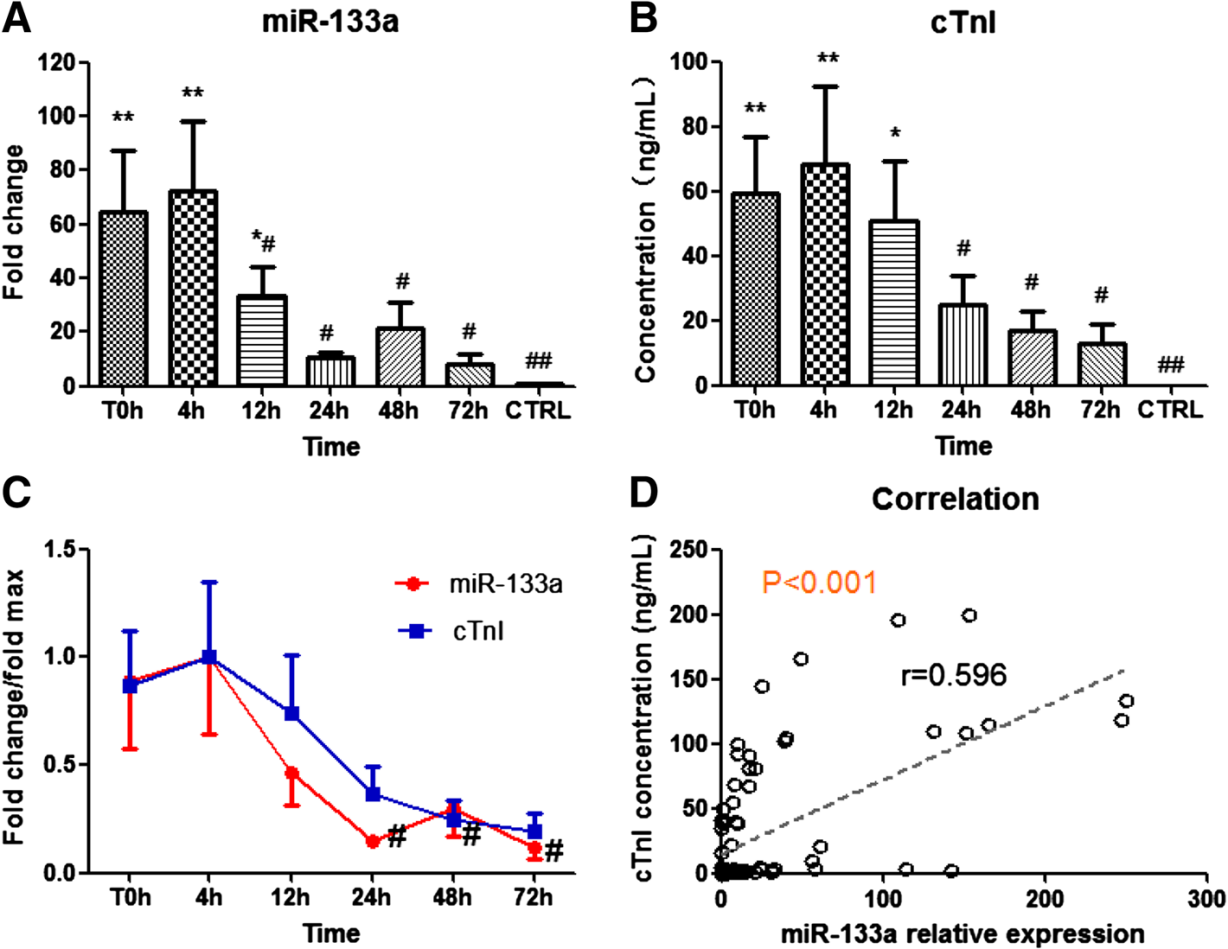
**Expression pattern of circulating miR-133a and cTnI in AMI patients. (A** and **B)** The expressions of circulating miR-133a and cTnI in AMI patients at various time points; **(C)** Time courses of circulating miR-133a and cTnI in AMI patients; **(D)** The correlation between miR-133a and cTnI in plasma from AMI patients. Data were presented as mean ± SEM, *p < 0.05, **p < 0.01 versus healthy control; ^#^p < 0.05, ^##^p < 0.01 versus peak expression (Statistical methods: ANOVA and linear regression analysis were used as appropriate).

Firstly, 13 AMI patients and 27 healthy volunteers were selected in the first cohort to test whether circulating miR-133a could serve as a novel biomarker of AMI. The baseline clinical characteristics of the first cohort are shown in Additional file 1: Table S1. There were no significant differences in age, sex, blood pressure, fasting glucose and other biochemical parameters between AMI patients and healthy volunteers. Six blood samples were obtained from each AMI patient at various time points (T0h, 4 h, 12 h, 24 h, 48 h, and 72 h) to investigate the dynamic change trend in circulating miR-133a level in the early phase of AMI. The first plasma sample was collected at 17.6 ± 4.5 hours after the onset of AMI symptoms (T0h), and other 5 collecting time points were 4 h, 12 h, 24 h, 48 h, and 72 h after T0. As shown in Figure 1A, circulating miR-133a concentrations were significantly increased in the early phase (the first 3 time points) after the occurrence of AMI in all patients compared with controls. To get a more intuitive look at the data, we also presented data using scatter plots (Additional file 1: Figure S3A). In those patients, circulating miR-133a achieved a peak (~72.1 fold) at 4 h, and showed a tendency to gradually return close to its control level over the next 3 days. The concentrations of cTnI were measured in the same blood samples from AMI patients, simultaneously. Interestingly, cTnI remarkably increased in the early phase of AMI and achieved a peak (~2445.2 fold) at 4 h resembling miR-133a (Figure 1B). They both exhibited the same trend in the early phase of AMI with the elevated peak at 4 h, and then gradually declined close to their normal level over the next 3 days (Figure 1C). Furthermore, correlation analysis showed a positive correlation between circulating levels of miR-133a and cTnI concentrations in AMI patients (Figure 1D). These data suggested that circulating miR-133a may be regarded as a novel biomarker of acute myocardial infarction.

### The correlation between plasma miR-133a levels and the severities of coronary lesion in CHD patients

**Figure 2:**
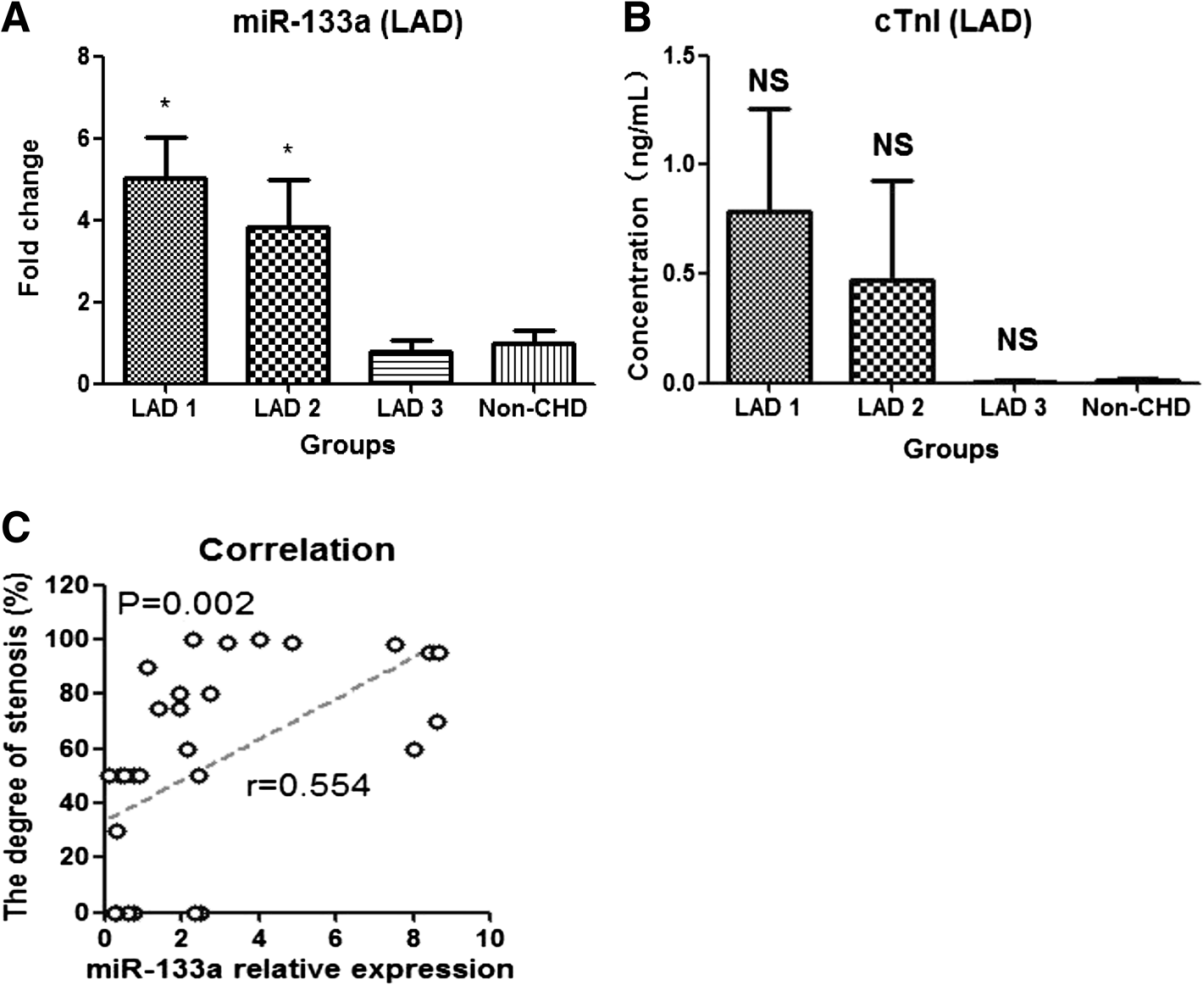
**Expression pattern of circulating miR-133a and cTnI in CHD patients with single stenosis in the proximal of left anterior descending coronary artery. (A** and **B)** The expressions of circulating miR-133a and cTnI in CHD patients with single stenosis of coronary artery; **(C)** The correlation between plasma miR-133a and the degree of coronary atherosclerotic stenosis in CHD patients with single stenosis in the proximal of left anterior descending coronary artery. Data were presented as mean ± SEM, *p < 0.05, **p < 0.01, NS, not significant versus non-CHD chest pain patient (Statistical methods: Student’s *t* test and linear regression analysis were used as appropriate).

To investigate whether circulating miR-133a expression correlates with the severity of coronary artery stenosis in CHD patients, 22 CHD patients with single stenotic lesion in the proximal left anterior descending coronary artery and 8 non-CHD patients with negative results of coronary angiography were recruited in the second cohort. Based on the severity of coronary artery stenosis, the participants were divided into 4 groups (Additional file 1: Table S2). The first group consisted of 8 CHD patients with severe coronary artery stenosis (81% ~ 100%, LAD 1); the second group contained 7 CHD patients with moderate coronary artery stenosis (51% ~ 80%, LAD 2); the third group consisted of 7 CHD patients with mild coronary artery stenosis (30% ~ 50%, LAD 3); the fourth group included 8 non-CHD patients with negative results of coronary angiography (non-CHD). Their characteristics are summarized in Additional file 1: Table S3. MiR-133a quantitative analysis showed that circulating miR-133a levels were significantly elevated in both LAD 1 and LAD 2 groups, especially in LAD 1 (~5-fold), compared to non-CHD patients, and the levels of miR-133a in LAD 3 showed no significant difference compared with non-CHD patients (Figure 2A and Additional file 1: Figure S3B). The concentration of plasma cTnI was not significantly different among the subgroups, although it was slightly higher in the first and second groups (Figure 2B). Linear regression analysis showed that the levels of plasma miR-133a positively correlated with the severity of coronary artery stenosis in CHD patients with single left anterior descending coronary atherosclerosis (Figure 2C), but there was no association between plasma cTnI and the degree of coronary stenosis. These results indicated that elevated miR-133a in plasma was better than cTnI for reflecting the severity of coronary artery stenosis in non-AMI CHD patients with single stenotic lesion of left anterior descending coronary artery.

### The correlation between circulating miR-133a levels and the gensini score in a validation cohort with a large number of CHD patients

**Figure 3:**
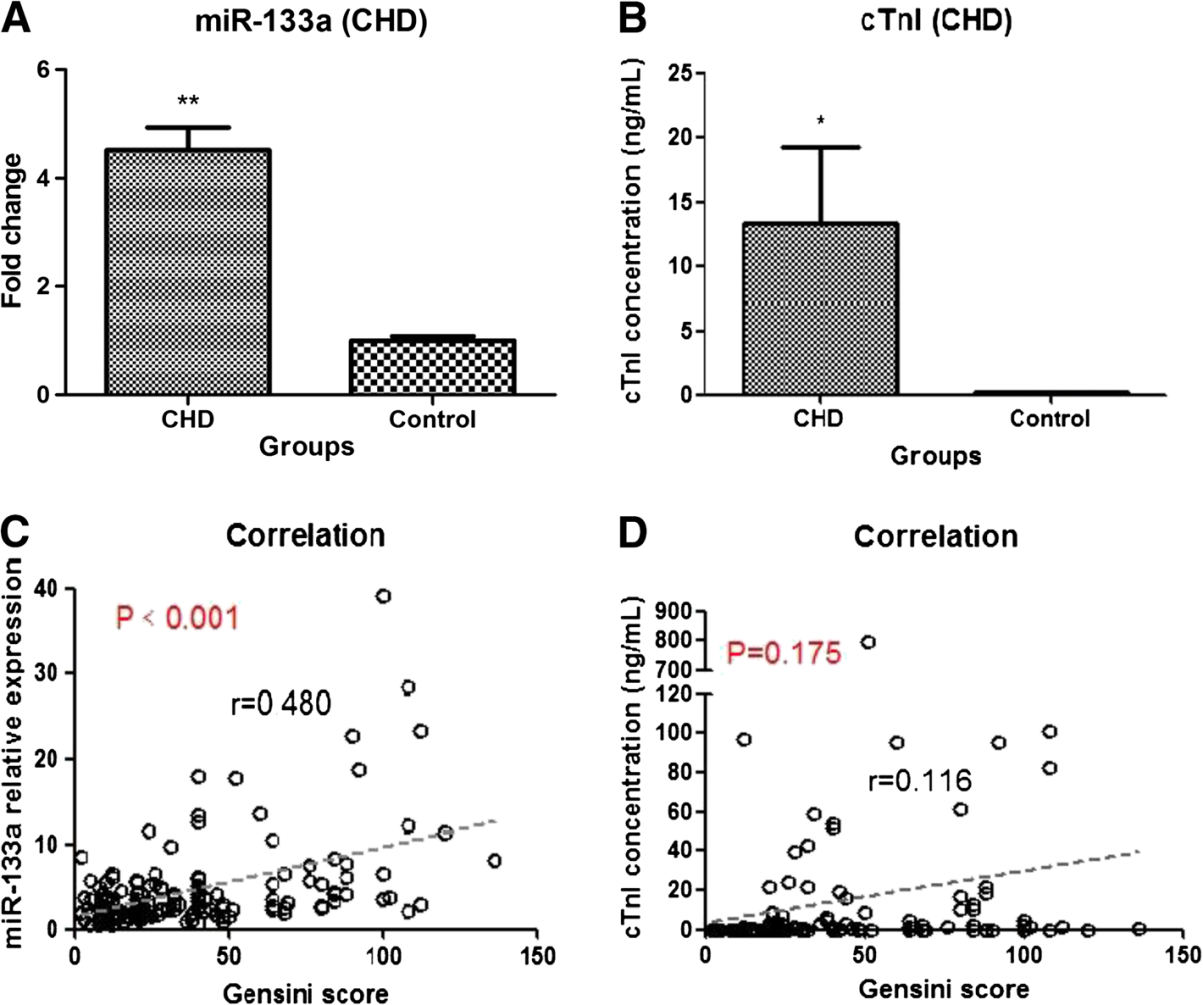
**Expression pattern of circulating miR-133a and cTnI in validation cohort of CHD patients. (A** and **B)** The expressions of circulating miR-133a and cTnI in CHD patients with complex stenosis of coronary artery; **(C** and **D)** The correlation between circulating miR-133a, cTnI and the Gensini scores in total CHD patients. Data were presented as mean ± SEM, *p < 0.05, **p < 0.01 versus non-CHD chest pain patient (Statistical methods: Student’s *t* test and linear regression analysis were used as appropriate).

**Figure 4:**
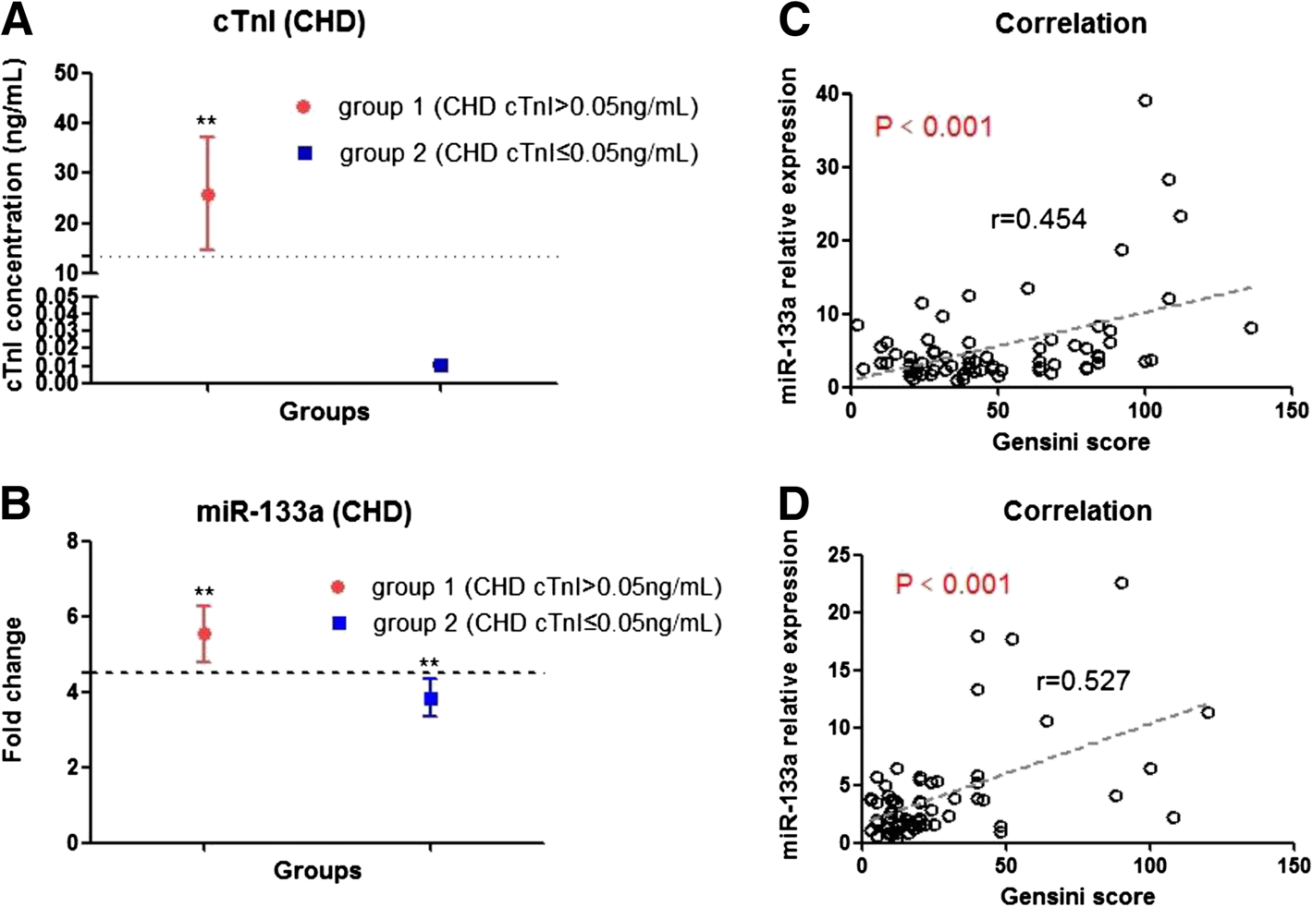
**Expression pattern of circulating miR-133a and cTnI in subgroups of CHD patients. (A** and **B)** The expressions of cTnI and circulating miR-133a in subgroups of CHD patients with complex stenosis of coronary artery; **(C** and **D)** The correlation between circulating miR-133a and the Gensini scores in patients from group 1 and group 2; Data were presented as mean ± SEM, *p < 0.05, **p < 0.01 versus non-CHD chest pain patient (Statistical methods: Student’s *t* test and linear regression analysis were used as appropriate).

The results obtained in the second cohort were then validated in a third cohort of 246 patients with acute chest pain. Among them, 154 patients had angiographically documented CHD, and the remaining 92 patients without evidence of CHD were selected as control (non-CHD patients). The baseline characteristics of this cohort are presented in Additional file 1: Table S4. All of these 154 CHD patients were assessed by Gensini score in order to evaluate the severity of coronary lesions. Our results showed a 4.5 fold increase of circulating miR-133a levels and an 87 fold increase of plasma cTnI concentrations in CHD patients compared with non-CHD chest pain patients, respectively (Figure 3A and B), and scatter plots on miR-133a levels more clearly illustrated these differences and distribution (Additional file 1: Figure S3C). Using the linear regression model, the relationship between circulating miR-133a (or cTnI) and Gensini score in 154 CHD patients were analyzed. The results showed that the levels of circulating miR-133a moderately correlated with Gensini scores in CHD patients (Figure 3C), while plasma cTnI did not show any correlation with Gensini scores (Figure 3D). Further, the plasma cTnI concentrations from 140 of 154 CHD patients were detected simultaneously. 72 of these 140 CHD patients had high levels of plasma cTnI, approximately 169-fold increase as compared with non-CHD patients (Figure 4A); but the remaining 68 CHD patients had very low levels of cTnI (≤ 0.05 ng/mL), which were not consistent with the severity of coronary artery stenosis. Further, in order to determine whether the expression of circulating miR-133a in CHD patients is superior to cTnI in the detection of the severity of coronary artery stenosis, 140 CHD patients were divided into 2 subgroups on the basis of cTnI levels (Figure 4A). The plasma miR-133a level increased ~5.5-fold and ~3.8-fold in group 1 and group 2, respectively, compared with non-CHD patients (Figure 4B). Moreover, in subgroup analysis, we found that plasma miR-133a level significantly correlated with Gensini score of coronary artery lesions in both subgroups of CHD patients (Figure 4C and D). However, the concentration of cTnI showed no correlation with the Gensini score in the two subgroups. These observations demonstrated that plasma miR-133a significantly correlated with the Gensini score of coronary lesions in CHD patients.

### The plasma miR-133a is a sensitive predictor for coronary heart disease

**Figure 5:**
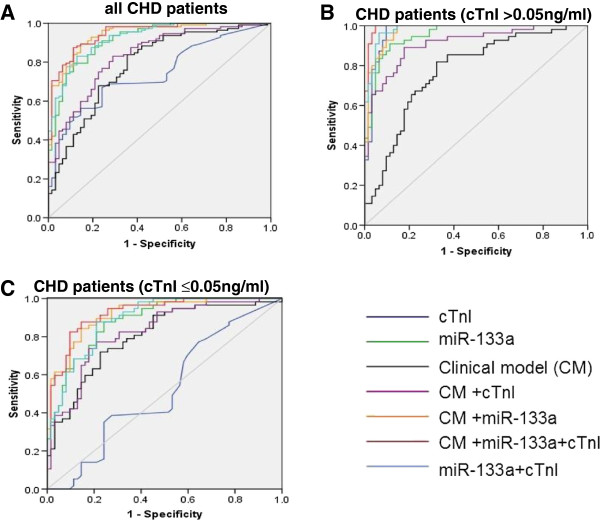
**Diagnostic value of cardiac troponin I and circulating miR-133a in CHD patients. (A)** Total CHD patients compared to non-CHD patients in the third cohort; **(B)** CHD patients from group 1 compared to non-CHD patients in the third cohort; **(C)** CHD patients from group 2 compared to non-CHD patients in the third cohort (Statistical methods: receiver operating characteristic (ROC) curve and multiple logistic regression analysis).

To investigate the role of circulating miR-133a as a sensitive predictor for CHD, ROC analysis was performed in the third cohort (Additional file 1: Table S5). The clinical model with age, sex, smoke and other cardiovascular risk factors (hypertension, diabetes, hyperlipidemia etc.) resulted in an AUC of 0.785 (95% confidence interval 0.713-0.857) for the third cohort to differentiate between CHD and non-CHD groups (Figure 5A). The ROC curve of cTnI showed a moderate separation, with an area under the ROC curve (AUC) of 0.741 (95% confidence interval 0.668-0.814). Interestingly, ROC curve of circulating miR-133a showed a much higher AUC of 0.918 (95% confidence interval 0.877-0.960). It is noteworthy that addition of miR-133a to the clinical model and cTnI remarkably increased the diagnostic value for CHD with an AUC of 0.942 (95% confidence interval 0.908-0.976) and 0.925 (95% confidence interval 0.887-0.963), respectively. Adding miR-133a to the clinical model with cTnI (AUC of 0.834, 95% confidence interval 0.773-0.896) significantly increased the AUC to an even higher value of 0.947 (95% confidence interval 0.915-0.979). Furthermore, the ROC curves of circulating miR-133a in both the subgroups of CHD were determined (Additional file 1: Table S6). MiR-133a also had significant differentiation value for CHD and non-CHD in both group 1 and group 2 with the AUC of 0.981 (95% confidence interval 0.962-1.0) and 0.885 (95% confidence interval 0.827-0.942), respectively (Figure 5B and C). The diagnostic value for CHD was significantly increased with the addition of miR-133a to cTnI in the two subgroups with the AUC of 0.953 (95% confidence interval 0.919-0.987) and 0.892 (95% confidence interval 0.836-0.947), respectively. Taken together, these data suggested that circulating miR-133a may be a sensitive and independent predictor for CHD.

## Discussion

Previous studies demonstrated that miRNAs are abundantly present in a remarkably stable form and they can be detected in peripheral circulation [12,31]. Recently, more and more circulating miRNAs, including heart-, vascular- and muscle-specific miRNAs, have been reported as new biomarkers in multiple cardiovascular diseases [32,33]. For example, circulating miR-423-5p is suggested as a biomarker for heart failure [34]. And additionally, cardiac-related miRNAs (miR-208, miR-499 and miR-1) and stress-related miRNAs (miR-21 and miR-146a) may be potential biomarkers for acute coronary syndrome [30]. Moreover, a recent study had reported that circulating miR-126, miR-223 and miR-197 were consistently and significantly related to incidence of myocardial infarction [35]. These observations suggest that circulating miRNAs may be useful not only for prediction of cardiovascular events, but also serve as sensitive biomarkers for improving the diagnostic accuracy of cardiovascular diseases.

The present study demonstrated dynamic change in circulating miR-133a expression in the early phase of acute myocardial infarction. Furthermore, our data is the first to demonstrate a positive correlation between circulating miR-133a and the severity of coronary stenosis in CHD patients.

The results demonstrated that circulating miR-133a levels increased in time-dependent manner in the early phase of AMI and exhibited a similar trend as cTnI in AMI patients; both of them rapidly increased at first, achieved a peak at 21.6 ± 4.5 hours after the onset of AMI symptoms, and then gradually returned close to normal level on the following days. Importantly, the circulating miR-133a positively correlated with cTnI in AMI patients. These results strongly indicated that circulating miR-133a can be a biomarker for diagnosing acute myocardial infarction.

Furthermore, 22 CHD patients with single lesion of coronary artery were included in the second cohort to study the relationship between plasma miR-133a and the severity of coronary atherosclerosis. The results showed that circulating miR-133a increased in CHD patients compared with non-CHD patients, and the levels of elevated miR-133a positively correlated with the severities of coronary atherosclerosis. The results were further verified in a large validation cohort of 246 subjects (154 CHD patients and 92 non-CHD). Interestingly, we found a higher expression of circulating miR-133a in CHD patients with low cTnI expression compared with non-CHD patients and it correlated with Gensini score of these CHD patients. These results showed that circulating miR-133a is superior to cTnI in detecting the severity of coronary artery lesions.

Finally, the ROC curve of miR-133a and cTnI were plotted in CHD patients with an AUC of 0.918 and 0.741, respectively. The ROC curves of CHD subcategories revealed that circulating miR-133a is more informative for CHD diagnosis than cTnI in CHD patients. Importantly, the diagnostic accuracy for CHD became significantly raised when combining clinical model, miR-133a and cTnI with the AUC of 0.947. Interestingly, this addition effect of combination could be more valuable for cTnI to improve the diagnostic accuracy of CHD, while miR-133a appeared to be a strong and independent predictor for CHD. These results may provide theoretical foundation in improving the clinical diagnosis of CHD.

In summary, the present study measured the early changes in expressions of circulating miR-133a in AMI patients, and provided first insights into the relationship between plasma miR-133a and the severity of coronary atherosclerotic stenosis in CHD patients, our results suggested that circulating miR-133a was a sensitive predictor for diagnosing AMI and CHD.

All these results suggested that circulating miR-133a can be a novel and potent biomarker for CHD, especially for AMI. And its level in plasma can reflect the severity of coronary atherosclerosis in CHD patients.

## Competing interests

The authors declare that they have no competing interests.

## Authors’ contributions

FW and GL carried out the miRNA detection studies and performed the statistical analysis. CZ and HL participated in collecting blood samples. SC and YW helped in drafting the manuscript. FW, CC and DWW conceived of the study, participated in its designing and writing the manuscript. All authors read and approved the final manuscript.

## Supplementary Material

Additional file 1: Figure S1 The qRT-PCR amplification curves and melting curves for both miR-133a and U6. **Figure S2.** The agarose gel electrophoresis images for both (A) miR-133a and (B) U6. RNA extract from mouse tissue (heart and brain). **Figure S3.** miR-133a expression in three cohorts displayed by scatter. **Table S1.** The clinical characteristics of 13 AMI patients and 27 healthy volunteers. **Table S2.** Divide the second cohort into 4 groups according to the degree of coronary artery stenosis. **Table S3.** The clinical characteristics of 22 CHD patients and 8 non-CHD patients. **Table S4.** The clinical characteristics of 154 CHD patients and 92 non-CHD patients. **Table S5.** Diagnostic value of cTnI and miR-133a in CHD patients in a clinical model. **Table S6.** Diagnostic value of cTnI and miR-133a in subgroups of CHD patients in a clinical model. Click here for file

## References

[B1] White HD Chew DP Acute myocardial infarction Lancet 2008 372 570 584 10.1016/S0140-6736(08)61237-4 18707987

[B2] Jaffe AS Ravkilde J Roberts R Naslund U Apple FS Galvani M Katus H It’s time for a change to a troponin standard Circulation 2000 102 1216 1220 10.1161/01.CIR.102.11.1216 10982533

[B3] Abbas NA John RI Webb MC Kempson ME Potter AN Price CP Vickery S Lamb EJ Cardiac troponins and renal function in nondialysis patients with chronic kidney disease Clin Chem 2005 51 2059 2066 10.1373/clinchem.2005.055665 16166165

[B4] Finsterer J Stollberger C Krugluger W Cardiac and noncardiac, particularly neuromuscular, disease with troponin-T positivity Neth J Med 2007 65 289 295 17890788

[B5] Giannitsis E Katus HA Cardiac troponin level elevations not related to acute coronary syndromes Nat Rev Cardiol 2013 10.1038/nrcardio.2013.129 10.1038/nrcardio.2013.129 23979214

[B6] Rosjo H Varpula M Hagve TA Karlsson S Ruokonen E Pettila V Omland T Group FS Circulating high sensitivity troponin T in severe sepsis and septic shock: distribution, associated factors, and relation to outcome Intensive Care Med 2011 37 77 85 10.1007/s00134-010-2051-x 20938765 PMC3020309

[B7] Bartel DP MicroRNAs: genomics, biogenesis, mechanism, and function Cell 2004 116 281 297 10.1016/S0092-8674(04)00045-5 14744438

[B8] van Rooij E Olson EN MicroRNAs: powerful new regulators of heart disease and provocative therapeutic targets J Clin Invest 2007 117 2369 2376 10.1172/JCI33099 17786230 PMC1952642

[B9] Croce CM Oncogenes and cancer N Engl J Med 2008 358 502 511 10.1056/NEJMra072367 18234754

[B10] van Rooij E Marshall WS Olson EN Toward microRNA-based therapeutics for heart disease: the sense in antisense Circ Res 2008 103 919 928 10.1161/CIRCRESAHA.108.183426 18948630 PMC2725407

[B11] Kajimoto K Naraba H Iwai N MicroRNA and 3T3-L1 pre-adipocyte differentiation RNA 2006 12 1626 1632 10.1261/rna.7228806 16870994 PMC1557704

[B12] Mitchell PS Parkin RK Kroh EM Fritz BR Wyman SK Pogosova-Agadjanyan EL Peterson A Noteboom J O'Briant KC Allen A Circulating microRNAs as stable blood-based markers for cancer detection Proc Natl Acad Sci U S A 2008 105 10513 10518 10.1073/pnas.0804549105 18663219 PMC2492472

[B13] Skog J Wurdinger T van Rijn S Meijer DH Gainche L Sena-Esteves M Curry WT Jr Carter BS Krichevsky AM Breakefield XO Glioblastoma microvesicles transport RNA and proteins that promote tumour growth and provide diagnostic biomarkers Nat Cell Biol 2008 10 1470 1476 10.1038/ncb1800 19011622 PMC3423894

[B14] Chim SS Shing TK Hung EC Leung TY Lau TK Chiu RW Lo YM Detection and characterization of placental microRNAs in maternal plasma Clin Chem 2008 54 482 490 10.1373/clinchem.2007.097972 18218722

[B15] Wang GK Zhu JQ Zhang JT Li Q Li Y He J Qin YW Jing Q Circulating microRNA: a novel potential biomarker for early diagnosis of acute myocardial infarction in humans Eur Heart J 2010 31 659 666 10.1093/eurheartj/ehq013 20159880

[B16] Chen JF Mandel EM Thomson JM Wu Q Callis TE Hammond SM Conlon FL Wang DZ The role of microRNA-1 and microRNA-133 in skeletal muscle proliferation and differentiation Nat Genet 2006 38 228 233 10.1038/ng1725 16380711 PMC2538576

[B17] Fichtlscherer S De Rosa S Fox H Schwietz T Fischer A Liebetrau C Weber M Hamm CW Roxe T Muller-Ardogan M Circulating microRNAs in patients with coronary artery disease Circ Res 2010 107 677 684 10.1161/CIRCRESAHA.109.215566 20595655

[B18] Liu J Hao DD Zhang JS Zhu YC Hydrogen sulphide inhibits cardiomyocyte hypertrophy by up-regulating miR-133a Biochem Biophys Res Commun 2011 413 342 347 10.1016/j.bbrc.2011.08.101 21893044

[B19] Care A Catalucci D Felicetti F Bonci D Addario A Gallo P Bang ML Segnalini P Gu Y Dalton ND MicroRNA-133 controls cardiac hypertrophy Nat Med 2007 13 613 618 10.1038/nm1582 17468766

[B20] Li Q Lin X Yang X Chang J NFATc4 is negatively regulated in miR-133a-mediated cardiomyocyte hypertrophic repression Am J Physiol Heart Circ Physiol 2010 298 H1340 H1347 10.1152/ajpheart.00592.2009 20173049 PMC3774484

[B21] Luo J Cai Q Wang W Huang H Zeng H He W Deng W Yu H Chan E Ng CF A microRNA-7 binding site polymorphism in HOXB5 leads to differential gene expression in bladder cancer PLoS One 2012 7 e40127 10.1371/journal.pone.0040127 22768238 PMC3387002

[B22] Liu N Bezprozvannaya S Williams AH Qi X Richardson JA Bassel-Duby R Olson EN microRNA-133a regulates cardiomyocyte proliferation and suppresses smooth muscle gene expression in the heart Genes Dev 2008 22 3242 3254 10.1101/gad.1738708 19015276 PMC2600761

[B23] He B Xiao J Ren AJ Zhang YF Zhang H Chen M Xie B Gao XG Wang YW Role of miR-1 and miR-133a in myocardial ischemic postconditioning J Biomed Sci 2011 18 22 10.1186/1423-0127-18-22 21406115 PMC3066105

[B24] D'Alessandra Y Devanna P Limana F Straino S Di Carlo A Brambilla PG Rubino M Carena MC Spazzafumo L De Simone M Circulating microRNAs are new and sensitive biomarkers of myocardial infarction Eur Heart J 2010 31 2765 2773 10.1093/eurheartj/ehq167 20534597 PMC2980809

[B25] Kuwabara Y Ono K Horie T Nishi H Nagao K Kinoshita M Watanabe S Baba O Kojima Y Shizuta S Increased microRNA-1 and microRNA-133a levels in serum of patients with cardiovascular disease indicate myocardial damage Circ Cardiovasc Genet 2011 4 446 454 10.1161/CIRCGENETICS.110.958975 21642241

[B26] Thygesen K Alpert JS Jaffe AS Simoons ML Chaitman BR White HD Katus HA Lindahl B Morrow DA Joint ESC/ACCF/AHA/WHF Task Force for the Universal Definition of Myocardial Third universal definition of myocardial infarction Circulation 2012 126 2020 2035 10.1161/CIR.0b013e31826e1058 22923432

[B27] Long G Wang F Duan Q Chen F Yang S Gong W Wang Y Chen C Wang DW Human circulating microRNA-1 and microRNA-126 as potential novel indicators for acute myocardial infarction Int J Biol Sci 2012 8 811 818 22719221 10.7150/ijbs.4439 PMC3372885

[B28] Wang E Nie Y Zhao Q Wang W Huang J Liao Z Zhang H Hu S Zheng Z Circulating miRNAs reflect early myocardial injury and recovery after heart transplantation J Cardiothorac Surg 2013 8 165 10.1186/1749-8090-8-165 23816326 PMC3716980

[B29] Brase JC Johannes M Schlomm T Falth M Haese A Steuber T Beissbarth T Kuner R Sultmann H Circulating miRNAs are correlated with tumor progression in prostate cancer Int J Cancer 2011 128 608 616 10.1002/ijc.25376 20473869

[B30] Oerlemans MI Mosterd A Dekker MS de Vrey EA van Mil A Pasterkamp G Doevendans PA Hoes AW Sluijter JP Early assessment of acute coronary syndromes in the emergency department: the potential diagnostic value of circulating microRNAs EMBO Mol Med 2012 4 1176 1185 10.1002/emmm.201201749 23023917 PMC3494874

[B31] Gilad S Meiri E Yogev Y Benjamin S Lebanony D Yerushalmi N Benjamin H Kushnir M Cholakh H Melamed N Serum microRNAs are promising novel biomarkers PLoS One 2008 3 e3148 10.1371/journal.pone.0003148 18773077 PMC2519789

[B32] Adachi T Nakanishi M Otsuka Y Nishimura K Hirokawa G Goto Y Nonogi H Iwai N Plasma microRNA 499 as a biomarker of acute myocardial infarction Clin Chem 2010 56 1183 1185 10.1373/clinchem.2010.144121 20395621

[B33] Ai J Zhang R Li Y Pu J Lu Y Jiao J Li K Yu B Li Z Wang R Circulating microRNA-1 as a potential novel biomarker for acute myocardial infarction Biochem Biophys Res Commun 2010 391 73 77 10.1016/j.bbrc.2009.11.005 19896465

[B34] Tijsen AJ Creemers EE Moerland PD de Windt LJ van der Wal AC Kok WE Pinto YM MiR423-5p as a circulating biomarker for heart failure Circ Res 2010 106 1035 1039 10.1161/CIRCRESAHA.110.218297 20185794

[B35] Zampetaki A Willeit P Tilling L Drozdov I Prokopi M Renard JM Mayr A Weger S Schett G Shah A Prospective study on circulating MicroRNAs and risk of myocardial infarction J Am Coll Cardiol 2012 60 290 299 10.1016/j.jacc.2012.03.056 22813605

