# Supplementary material for: Plasma microRNA-133a is a new marker for both acute myocardial infarction and underlying coronary artery stenosis
Source: J Transl Med. 2013 Sep 23;11:222. doi: 10.1186/1479-5876-11-222 (PMC3849017; doi:10.1186/1479-5876-11-222)
Supplement: Additional file 1: Figure S1 — The qRT-PCR amplification curves and melting curves for both miR-133a and U6. Figure S2. The agarose gel electrophoresis images for both (A) miR-133a and (B) U6. RNA extract from mouse tissue (heart and brain). Figure S3. miR-133a expression in three cohorts displayed by scatter. Table S1. The clinical characteristics of 13 AMI patients and 27 healthy volunteers. Table S2. Divide the second cohort into 4 groups according to the degree of coronary artery stenosis. Table S3. The clinical characteristics of 22 CHD patients and 8 non-CHD patients. Table S4. The clinical characteristics of 154 CHD patients and 92 non-CHD patients. Table S5. Diagnostic value of cTnI and miR-133a in CHD patients in a clinical model. Table S6. Diagnostic value of cTnI and miR-133a in subgroups of CHD patients in a clinical model. [file 1479-5876-11-222-S1.doc]

**Figure S1**

**
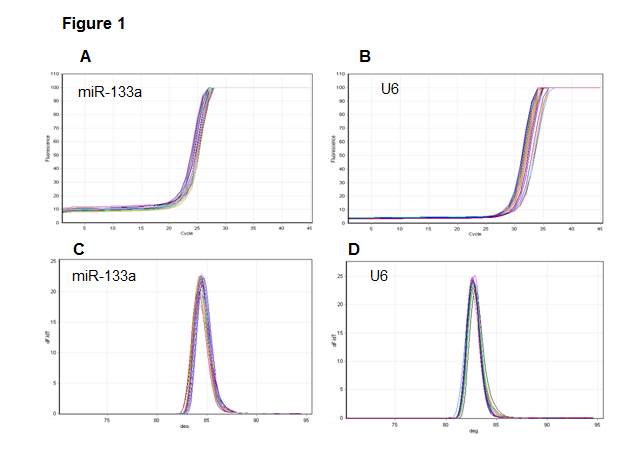
**

Figure S1. The qRT-PCR amplification curves and melting curves for both miR-133a and U6. (A and B) The qRT-PCR amplification curves of miR-133a and U6, respectively. (C and D) The qRT-PCR melting curves of miR-133a and U6, respectively.

**Figure S2**


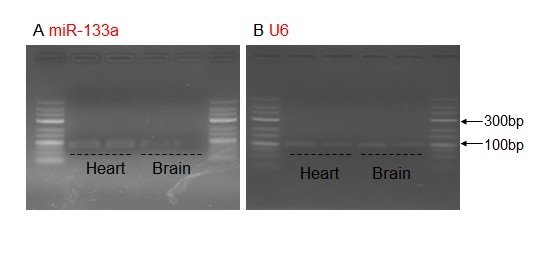


Figure S2. The agarose gel electrophoresis images for both (A) miR-133a and (B) U6. RNA extract from mouse tissue (heart and brain).

**Figure S3**


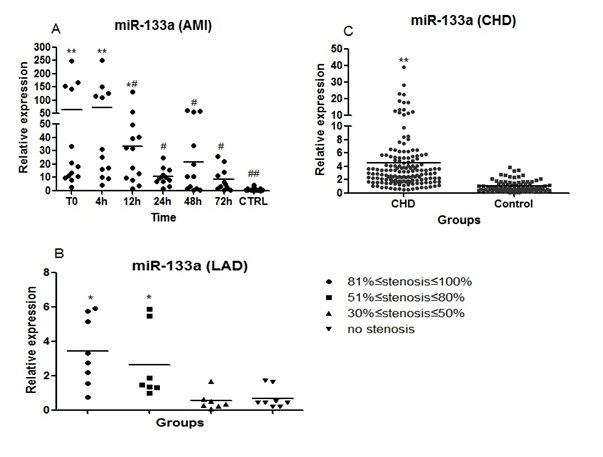


Figure S3. miR-133a expression in three cohorts displayed by scatter. Data were presented as mean, *p<0.05, **p<0.01 versus control; ^#^p<0.05, ^##^p<0.01 versus peak expression.

**Table S1: The clinical characteristics of 13 AMI patients and 27 healthy volunteers.**

| **Characteristics** | **Control (n=27)** | **AMI (n=13)** | **P value** |
| --- | --- | --- | --- |
| **Age (Years)** | 50.37±11.45 | 54.15±12.54 | 0.348 |
| **Male/Female (n/n)** | 13/14 | 9/4 | 0.209 |
| **BMI (kg/m)** | 27.5±6.9 | 23.8±10.6 | 0.325 |
| **SBP (mmHg)** | 126.1±15.7 | 127.8±13.0 | 0.738 |
| **DBP (mmHg)** | 76.1±12.0 | 80.4±12.7 | 0.309 |
| **GLU (mmol/L)** | 6.24±1.37 | 6.82±1.88 | 0.289 |
| **TG (mmol/L)** | 1.31±1.11 | 1.46±0.64 | 0.663 |
| **TC (mmol/L)** | 4.21±0.91 | 4.45±1.37 | 0.363 |
| **HDL (mmol/L)** | 1.17±0.25 | 1.04±0.22 | 0.125 |
| **LDL (mmol/L)** | 2.35±0.84 | 2.41±0.59 | 0.825 |

BMI indicates body mass index; SBP, systolic blood pressure; DBP, diastolic blood pressure; GLU, glucose; TG, Triglyceride; TC, total cholesterol; HDL, high-density lipoprotein; LDL, low-density lipoprotein; comparison between AMI patients and healthy persons.

**Table S2: Divide the second** cohort into 4 groups according to the degree of coronary artery stenosis

| **Groups** | **The extent of stenosis** | **The degree of stenosis** | **Number** |
| --- | --- | --- | --- |
| **LAD 1** | 81%≤stenosis≤100% | 97%±3.5% | 8 |
| **LAD 2** | 51%≤stenosis≤80% | 71.4%±8.5% | 7 |
| **LAD 3** | 30%≤stenosis≤50% | 47.1%±7.6% | 7 |
| **Non-CHD** | no stenosis | 0% | 8 |

CHD: coronary heart disease; Number: the number of each group

**Table S3: The clinical characteristics of 22 CHD patients and 8 healthy volunteers.**

| **Characteristic** | **LAD 1** | **LAD 2** | **LAD 3** | **Non-CHD** | **P value** |
| --- | --- | --- | --- | --- | --- |
| **Age (Years)** | 60.3±11.6 | 58.9±10.6 | 60.3±7.3 | 53.6±8.7 | 0.505 |
| **Male/Female (n/n)** | 6/2 | 4/3 | 6/1 | 5/3 | 0.641 |
| **BMI (kg/m)** | 29.74±4.8 | 32.19±6.3 | 32.73±3.6 | 31.06±11.0 | 0.876 |
| **SBP (mmHg)** | 125.1±18.0 | 131.4±12.7 | 140.3±23.9 | 116.7±14.7 | 0.096 |
| **DBP (mmHg)** | 77.9±15.2 | 81.3±13.9 | 82.6±12.8 | 69.4±6.2 | 0.184 |
| **GLU (mmol/L)** | 6.04±1.25 | 6.57±3.37 | 7.02±3.38 | 5.57±1.21 | 0.716 |
| **TG (mmol/L)** | 1.54±0.88 | 2.19±0.98 | 2.01±1.40 | 2.14±2.17 | 0.877 |
| **TC (mmol/L)** | 4.05±0.96 | 3.77±0.38 | 4.70±1.16 | 4.85±0.78 | 0.144 |
| **HDL (mmol/L)** | 0.93±0.21 | 1.05±0.17 | 1.07±0.21 | 1.34±0.12 | 0.005 |
| **LDL (mmol/L)** | 2.66±0.65 | 2.06±0.32 | 2.57±0.72 | 2.82±0.65 | 0.234 |

BMI indicates body mass index; SBP, systolic blood pressure; DBP, diastolic blood pressure; GLU, glucose; TG, total glyceride; TC, total cholesterol; HDL, high-density lipoprotein; LDL, low-density lipoprotein; comparison between CHD patients and non-CHD patients.

**Table S4: The clinical characteristics of 154 CHD patients and 92 non-CHD patients.**

| **Characteristics** | **CHD (n=154)** | **Non-CHD (n=92)** | **P value** |
| --- | --- | --- | --- |
| **Age (Years)** | 59.62±10.48 | 56.47±10.35 | 0.024 |
| **Male/Female (n/n)** | 120/34 | 45/47 | 0.001 |
| **BMI (kg/m)** | 24.26±3.60 | 23.82±2.89 | 0.278 |
| **SBP (mmHg)** | 132.2±21.5 | 129.6±18.9 | 0.336 |
| **DBP (mmHg)** | 79.7±14.3 | 80.3±12.6 | 0.764 |
| **GLU (mmol/L)** | 7.32±3.69 | 5.89±1.23 | 0.001 |
| **TG (mmol/L)** | 1.80±1.37 | 1.65±1.17 | 0.393 |
| **TC (mmol/L)** | 4.07±1.14 | 4.37±1.37 | 0.075 |
| **HDL (mmol/L)** | 1.14±1.03 | 1.11±0.26 | 0.846 |
| **LDL (mmol/L)** | 2.38±0.94 | 2.47±0.67 | 0.446 |

BMI indicates body mass index; SBP, systolic blood pressure; DBP, diastolic blood pressure; GLU, glucose; TG, total glyceride; TC, total cholesterol; HDL, high-density lipoprotein; LDL, low-density lipoprotein; comparison between CHD patients and non-CHD patients.

**Table S**5: Diagnostic value of cTnI and miR-133a in CHD patients in a clinical model.

| **Marker** | **AUC** | **95% Cl** |
| --- | --- | --- |
| Clinical model(CM) | 0.785 | 0.713-0.857 |
| cTnI | 0.741 | 0.668-0.814 |
| miR-133a | 0.918 | 0.877-0.960 |
| miR-133a+cTnI | 0.925 | 0.887-0.963 |
| CM+cTnI | 0.834 | 0.773-0.896 |
| CM+miR-133a | 0.942 | 0.908-0.976 |
| CM+cTnI+miR-133a | 0.947 | 0.915-0.979 |

CM, clinical model of CHD patients (including age, sex, BMI, smoke, hypertension, diabetes, hypercholesterolemia); cTnI, Cardiac troponin I; miR-133a, microRNA-133a; AUC, area under the ROC curve; 95% Cl, 95% confidence interval.

**Table S**6: Diagnostic value of cTnI and miR-133a in subgroups of CHD patients in a clinical model.

| **Group 1 (cTnI>0.05ng/L)** | | | **Group 2 (cTnI≤0.05ng/L)** | | |
| --- | --- | --- | --- | --- | --- |
| **Marker** | **AUC** | **95% Cl** | **Marker** | **AUC** | **95% Cl** |
| CM | 0.774 | 0.689-0.859 | CM | 0.802 | 0.723-0.881 |
| cTnI | 0.970 | 0.940-1.000 | cTnI | 0.520 | 0.415-0.625 |
| miR-133a | 0.953 | 0.919-0.987 | miR-133a | 0.885 | 0.827-0.942 |
| miR-133a+cTnI | 0.981 | 0.962-1.000 | miR-133a+cTnI | 0.892 | 0.826-0.947 |
| CM+cTnI | 0.911 | 0.858-0.963 | CM+cTnI | 0.826 | 0.752-0.900 |
| CM+miR-133a | 0.974 | 0.950-0.998 | CM+miR-133a | 0.919 | 0.871-0.966 |
| CM+cTnI+miR-133a | 0.993 | 0.000-1.000 | CM+cTnI+miR-133a | 0.925 | 0.879-0.971 |

CM, clinical model of CHD patients (including age, sex, BMI, smoke, hypertension, diabetes, hypercholesterolemia); cTnI, Cardiac troponin I; miR-133a, microRNA-133a; AUC, area under the ROC curve; 95% Cl, 95% confidence interval.
